# Bacterial Meningoencephalitis in Newborns

**DOI:** 10.3390/biomedicines12112490

**Published:** 2024-10-30

**Authors:** Alessia Guarnera, Giulia Moltoni, Francesco Dellepiane, Giulia Lucignani, Maria Camilla Rossi-Espagnet, Francesca Campi, Cinzia Auriti, Daniela Longo

**Affiliations:** 1 Functional and Interventional Neuroradiology Unit, Bambino Gesù Children’s Hospital, IRCCS, 00165 Rome, Italy; giulia.moltoni@opbg.net (G.M.); francesco.dellepiane@opbg.net (F.D.); giulia.lucignani@opbg.net (G.L.); mcamilla.rossi@opbg.net (M.C.R.-E.); daniela.longo@opbg.net (D.L.); 2 Neuroradiology Unit, NESMOS Department Sant’Andrea Hospital, La Sapienza University, Via di Grottarossa, 1035-1039, 00189 Rome, Italy; 3 Neonatal Intensive Care Unit, Department of Medical and Surgical Neonatology, Bambino Gesù Children’s Hospital, IRCCS, 00146 Rome, Italy; francesca.campi@opbg.net (F.C.); cinzia.auriti@gmail.com (C.A.)

**Keywords:** bacterial meningoencephalitis, meningoencephalitis, meningitis, encephalitis, pediatric, newborns, MRI, US, CT

## Abstract

Bacterial meningoencephalitis in newborns is a severe and life-threatening pathology, which results from meningeal infection and the subsequent involvement of the brain parenchyma. The severity of the acute onset of symptoms and the risk of neurodevelopmental adverse sequelae in children strongly depend on the timing of the infection, the immunological protection transmitted by the mother to the fetus during pregnancy, and the neonate’s inflammatory and immune system response after birth. Although the incidence of neonatal meningitis and meningoencephalitis and related mortality declined in the past twenty years with the improvement of prenatal care and with the introduction of intrapartum antibiotic prophylaxis against Streptococcus beta Hemolyticus group B (Streptococcus Agalactiae) in the 1990s, bacterial meningitis remains the most common form of cerebrospinal fluid infection in pediatric patients. To date, the rate of unfavorable neurological outcomes is still from 20% to 60%, and the possibility of containing its rate strongly depends on early diagnosis, therapy, and a multidisciplinary approach, which involves neonatologists, neurologists, neuroradiologists, and physiotherapists. Neonatal meningitis remains difficult to diagnose because the responsible bacteria vary with gestational age at birth, age at presentation, and environmental context. The clinical presentation, especially in the newborn, is very ambiguous. From a clinical point of view, the definitive test for diagnosis is lumbar puncture in patients with symptoms suggestive of neurological involvement. Therefore, neuroimaging is key for raising clinical suspicion of meningitis or corroborating the diagnosis based on clinical and laboratory data. Our pictorial review offers a practical approach to neonatal meningoencephalitis by describing the epidemiology, the pathophysiology of bacterial meningoencephalitis, defining the indications and suggesting optimized protocols for neuroimaging techniques, and showing the main neuroimaging findings to reach the diagnosis and offering proper follow-up of bacterial meningitis. Moreover, we tried identifying some peculiar MRI patterns related to some bacteria.

## 1. Introduction

Bacterial meningitis and meningoencephalitis in newborns are severe and life-threatening pathologies, which result from meningeal infection and the subsequent involvement of the brain parenchyma. The severity of the acute onset of symptoms and the risk of neurodevelopmental adverse sequelae in childhood strongly depend on the timing of the infection, the immunological protection transmitted by the mother to the fetus during pregnancy, and the neonate’s inflammatory and immune system response after the birth [1,2]. Although the incidence of neonatal meningitis and meningoencephalitis and related mortality declined in the past twenty years with the improvement of prenatal care and with the introduction of intrapartum antibiotic prophylaxis against *Streptococcus beta Hemolyticus group B (Streptococcus Agalactiae)* in the 1990s, bacterial meningitis remains the most common form of cerebrospinal fluid infection in pediatric patients. To date, the rate of unfavorable neurological outcomes is still from 20% to 60%, and the possibility of containing its rate strongly depends on early diagnosis, therapy, and a multidisciplinary approach, which involves neonatologists, neurologists, neuroradiologists, and physiotherapists [3,4].

Neonatal meningitis remains difficult to diagnose because the responsible bacteria vary with gestational age at birth, age at presentation, and environmental context. The clinical presentation in the newborn is very ambiguous. Although the definitive test for diagnosis is lumbar puncture in patients with symptoms suggestive of neurological involvement, neuroimaging is key for raising clinical suspicion of meningitis or corroborating the diagnosis based on clinical and laboratory data. Ultrasound (US) is the first neuroimaging modality because it is cost-effective, rapid, and may be performed at the bedside. Magnetic Resonance Imaging (MRI) is the neuroradiological gold standard and offers a panoramic and extensive evaluation of the neonate’s intra-axial and extra-axial compartment. Nevertheless, sedation can be sometimes avoided thanks to the use of the feed and wrap method in newborns weighing more than four kilos [5]. CT is mainly limited to an emergency setting since it requires the use of radiation, and low-dose protocols are suggested for limiting the patient’s irradiation [2,6,7,8].

Objectives of this pictorial review are as follows: (a) to describe the epidemiology and pathophysiology of bacterial meningoencephalitis in neonates; (b) to suggest optimized protocols and indications for the US, CT, and MRI performance; (c) to show the main neuroimaging findings at diagnosis and follow-up; and (d) to describe some peculiar neuroimaging patterns related to specific bacteria.

## 2. Epidemiology

Neonatal meningitis has an incidence of 0.3/1000 live births in industrialized countries and despite the mortality decreasing from 50% in the 1970s to less than 10% in recent years, the neurological sequelae occur from 20% to 60% of patients [1,9,10].

Infectious bacterial meningitis is the most common form of cerebrospinal fluid (CSF) infection in neonates and children. The occurrence of meningitis in newborns is strongly related to neonatal sepsis, which is split into two categories in relation to the age of onset of symptoms after birth: sepsis that arises within 72 h of life is called early-onset sepsis, while the one occurring after 72 h is called late-onset sepsis. The exception is sepsis due to *Group B beta-hemolytic streptococcus*, which is considered an early-onset sepsis up to the first 7 days of life. Usually, 90% of neonatal sepsis occurs in the first 24 h of life [2,8,11].

The incidence of early-onset sepsis is from 0.5 to 8/1000 live births in term newborns and from 15 to 19/1000 live births in preterm patients weighing <1500 g. *Streptococcus Agalactiae* remains the most common cause of meningitis and neonatal sepsis, responsible for more than 40% of all early-onset infections. Meningitis complicates 5 to 20% of cases of early-onset sepsis [2,12]. Early-onset sepsis is related to maternal–fetal transmission during pregnancy or at delivery. The main neonatal risk factors are prematurity and low weight at birth. Maternal risk factors are represented by infections contracted during pregnancy, recto-vaginal colonization by *Group B Streptococcus beta-hemoliticus*, placentitis, chorioamnionitis, and the premature rupture of fetal membranes [13,14,15].

Late-onset sepsis is primarily attributed to nosocomial or horizontal pathogen acquisition and exposure to hospital or community environments. *Streptococcus Agalactiae* and *E. coli* remain the predominant causative bacteria for late community-acquired sepsis and meningitis. In neonates admitted to the NICU (Neonatal Intensive Care Unit) at the time of symptom onset, the most common causative bacteria are *Coagulase-negative Staphylococci* and *Staphylococcus Aureus*, followed by *E. coli and Klebsiella pneumonia*. Preterm birth and critical illness are major risk factors for late-onset sepsis due to the need for hospitalization and medical devices (i.e., central catheters, mechanical ventilation, and parenteral nutrition). Late-onset sepsis rates are reported at 1.6% in term neonates, compared to 12–50% among very preterm and/or very low birth weight infants. It is complicated by meningitis in approximately 5% of cases [16].

## 3. Pathogenesis

In newborns, the hematogenous spread of bacteria during sepsis causes them to enter the vessels of the choroid plexus of the brain, leading to the onset of meningitis [2]. The following plexitis and ventriculitis cause pathogen dissemination in the cerebrospinal fluid with the involvement of the leptomeningeal space and the subsequent diffusion of the inflammation to the arachnoid membrane. Arachnoiditis leads to the formation of membranes and adhesion, causing hydrocephalus. The seepage of the inflammatory exudate in the subdural space may lead to subdural effusions that may become infected and result in subdural empyemas [8,17,18,19].

**Figure 1:**
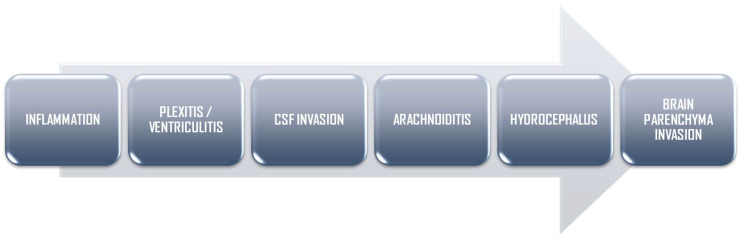
Flowchart illustrating the pathophysiology and progression of bacterial meningoencephalitis.

Brain parenchyma invasion is often secondary to meningeal invasion, leading to cerebritis, abscesses, and cerebral infarcts, which have been identified in up to 30% of neonates with bacterial meningitis [20,21,22]. The most common clinical symptoms are lethargy, stupor, poor feeding, and irritability, which are associated with seizures in 40% of cases. Neonates often present with bulging fontanels [8,23] (Figure 1).

Long-term sequelae of meningoencephalitis are encephalomalacia and gliosis coexisting with cortical and white matter atrophy, and persistent hydrocephalus.

## 4. Role of Neuroimaging and Neuroradiological Techniques

Meningitis is a severe pathology, and its diagnosis is typically made using data obtained through clinical and laboratory examination, particularly CSF analysis. Neuroimaging has a central role in raising the suspicion of meningoencephalitis and its complications, and monitoring the evolution during the follow-up since the neuroradiological findings vary widely ranging from nonspecific to peculiar findings depending on the disease stage. Moreover, imaging allows a safe lumbar puncture to be performed by excluding raised intracranial pressure [2,8].

### 4.1. US Technique

The first imaging modality is represented by the US, thanks to the presence of open fontanels in newborns. The US is a rapid, cost-effective, and safe exam, which does not involve the administration of ionizing radiation. It may be performed at the bedside without patient sedation and may be repeated over time.

US shows some limitations that should be mentioned. The most important limitations are that the technique is extremely operator-dependent, as the exam performance and interpretation depend on the competence and experience of the neuroradiologist performing the exam, and patient-dependent, as the newborns may show low compliance and the tutor’s or parent’s help might be needed to continue the exam. Moreover, it relies on open fontanels and does not allow an evaluation of the entire convexity. Finally, it is a first-level exam; thus, early and subtle neonatal meningitis may not be detectable and the US exam may appear totally normal.

The quality of the US is dependent on the competence and experience of the sonographer, yet there are some general indications that may offer a simple pipeline to perform an optimal neonatal US. Neonatal sonography should be performed with a high-frequency probe (>7.5 MHz) and proper depth and TGC (time and gain compensation), which should be optimized to produce an image filling the sector and containing cranial contours [6]. The most common approach is through the anterior fontanels. The complete evaluation of the infratentorial structures may be limited in relation to their location and the barrier caused by the echogenic tentorium. Additional and complementary approaches that offer alternative acoustic windows are the posterolateral fontanelle approach at the mastoid level, the lateral fontanel approach at the temporal level, the lambdoid fontanelle approach posteriorly, and the foramen magnum approach [6,24]. The acquired images should be symmetrical and at least five on the coronal and sagittal planes respectively. The sonographer should optimally evaluate the superficial structures, such as meninges, subdural space, and cortex. A Transcranial Doppler sonography investigation is essential for the evaluation of the vessel structure and patency. In particular, it is essential to evaluate the superior sagittal sinus in a coronal plane through an anterior fontanelle approach, the tangential vessels in the subarachnoid space, and the intracranial vessels. The Doppler box should be focused on the area of interest and used to evaluate flow velocities and indices, especially of the intracranial vessels. The Power Doppler should be used to evaluate small vessels, although it is still not possible to acquire any flow direction or other indices [6,24].

### 4.2. MRI Technique and Protocol

MRI is the imaging gold standard to diagnose meningitis and its complications since it allows a complete and extensive evaluation of intracranial structures. On the other hand, the main limitations of performing a brain MRI of newborns should be pointed out. Particularly, MRI exams require long acquisition times that may result in the need for neonate sedation and, therefore, for the presence of an expert neonatal anesthesiologist. To overcome this inconvenience, it may be used the feed and wrap technique to acquire newborn MRIs in spontaneous sleep. This technique consists of asking the mother to feed the neonate in a comfortable setting before the scan to induce natural sleep, followed by swaddling to reduce motion artifacts [5]. Furthermore, newborn brain MRI interpretation and reporting require highly qualified and trained personnel, and the cost of MRI scanner purchase and exam acquisition is extremely costly. Due to these reasons, MRI scanners are not always available in some institutions or emergency settings [8,23].

To date, there is no standardized MRI protocol for neonatal meningitis. The choice of MRI sequences should be accurately selected to perform high-quality exams in a short time since the neonate is often sedated by the anesthesiologist. The key sequences are pre-contrast T1WI (weighted imaging) and T2WI in at least two perpendicular planes or acquired as 3D sequences for the morphological evaluation; DWI (diffusion-weighted imaging)/ADC (apparent diffusion coefficient) for its high sensitivity and early detection of lesions and cerebral complications such as cerebral infarcts; SWI (susceptibility weighted imaging) and GRE (gradient-echo) T2*, which identify engorged and thrombotic vessels, small infarcts, and small hemorrhagic foci; and postcontrast 3D T1 MPRAGE (Magnetization Prepared Rapid Gradient Echo Imaging), which is among the pivotal sequences for evaluating meningeal and brain alterations. Among the additional sequences that have shown a significant impact on meningitis diagnosis and follow-up, we cite MRS (MR spectroscopy) to evaluate different molecular peaks, which may be extremely useful in the differential diagnosis between brain abscesses and tumors; 3D T1 TSE (turbo spin echo) BB (black-blood), which allows evaluating vessel wall abnormalities (ex. vessel wall thickening and enhancement) beyond luminal abnormalities, such the presence of cerebral arteritis; CE-MRV (contrast-enhanced MR venography) or, if contrast medium administration is not indicated, 2-D time-of-flight MR venography image for a proper evaluation of venous vessel; and T2 3D CISS (Constructive Interference in Steady State) or T2 3D SPACE sequences, especially in the follow-up for the evaluation of synechia or membranes causative of hydrocephalus. Before and after contrast administration, a FLAIR sequence can be used to depict meningeal and ventricular alterations related to infection and during follow-up. However, it is important to bear in mind that in newborns, the incomplete myelination process makes FLAIR often inappropriate for the evaluation of the brain parenchyma.

The suggested protocol of our Neuroradiology Unit at the Bambino Gesù Hospital in Rome, using a 3T scanner (Magnetom Skyra, Siemens, Erlangen, Germany) with a 32-channel brain coil (L-W-H: 440 mm × 330 mm × 370 mm), is reported in Table 1.

### 4.3. The Limited Role of CT and Low-Dose Protocols

CT is usually performed in a first-aid setting characterized by time restriction or in the absence of an MRI in some institutions. This choice is based on the lower specificity and sensitivity of CT compared to MRI and the disadvantage of radiation exposure. The main limitation related to CT acquisitions in newborns is radiation exposure. As a matter of fact, the choice of the neuroimaging technique should always be rooted in offering the best diagnostic tool to the patient and in following the ALARA (as low as reasonably achievable) principle in case of radiation [2,5]. Multiple efforts have been made to propose low-dose CT protocols, namely to obtain high-quality CT images with the least radiation possible, thanks to the help of Artificial Intelligence algorithms [7,25,26].

## 5. Bacterial Meningitis and Complications: Neuroradiological Findings (Table S1)

### 5.1. Choroid Plexitis and Ventriculitis

Ventricle involvement is a frequent complication of meningitis and is secondary to choroid plexitis, namely spread through the infected ventricle plexus, or is related to a parenchymal abscess rupture into the ventricular system.

Choroid plexitis is easily identifiable with the ultrasound technique as a hyperechogenic and irregular choroid plexus, while ventriculitis is characterized by a thickened ependyma showing increased echogenicity and focal irregularities, indicating focal ependymal loss resulting in glial proliferation. The associated subependymal inflammatory infiltration causes an increased echogenicity of the periventricular territories. Ventricles lose the typical anechogenic appearance and show low-level internal echoes in relation to inflammatory exudates that may organize into septa causing ventricular compartmentalization and secondary hydrocephalus [6,27,28].

**Figure 2:**
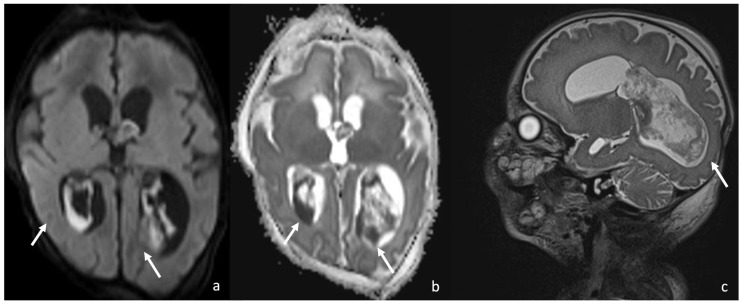
A 10-day-old boy with *Serratia Meningitis,* presenting with plexitis. Axial DWI (**a**) and axial ADC (**b**) show infection of the choroid plexus characterized by diffusion restriction (arrows in (**a**,**b**)), and sagittal T2 SPACE shows engorgement and infection of the choroid plexus (arrow in (**c**)).

**Figure 3:**
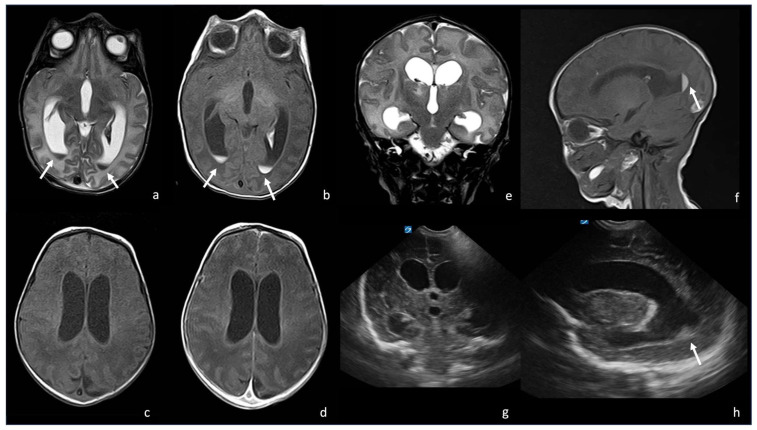
A 9-day-old boy affected by meningitis caused by *Listeria Monocytogenes* with ventriculitis. There are hypointense in T2 (arrow in (**a**), axial T2WI) and hyperintense detritus in T1 (arrow in (**b**), axial T1-weighted images); a slight contrast enhancement of ventricle wall is visible in (**d**), axial post- contrast T1 WI, especially if compared with (**c**) axial pre-contrast T1 WI. There is also a ventriculomegaly visible on both MRI ((**e**), coronal T2WI; (**f**), sagittal T1 WI) and US ((**g**), coronal plane and (**h**), parasagittal plane). In (**f**,**h**), there are also visible hyperintense ((**f**), arrow) and hyperechogenic detritus ((**h**), arrow).

Contrast-enhanced T1WI shows the engorgement of the choroid plexus and linear enhancement of the ependyma and is not able to provide a proper CSF absorption, leading to ventricular enlargement and subsequently to hydrocephalus. The CSF inside the ventricles is inhomogeneous and characterized by a level since proteinaceous debris and purulent tissue stratify in the dependent portions of the ventricles, more frequently in the occipital horns, which show restricted diffusion in DWI/ADC. FLAIR shows a slightly hyperintense signal of the dependent debris and purulent tissue inside the hypointense ventricles. Post-contrast T1WI may show the presence of subtle enhancing membranes, indicating intraventricular septa and fibrotic strands. The periventricular WM may show an abnormal signal in relation to necrosis, which is related to bacterial toxins or thrombosis of the subependymal and periventricular veins [8,29,30,31] (Figure 2 and Figure 3).

CT is inferior to MRI in diagnosing subtle meningitis, yet the most prevalent signs include enhancing empyema and ventricular enlargement.

At follow-up (FU) MRI, in the case of early diagnosis and optimal therapy, choroid plexa and ventricula appear normal at imaging. Among the most common chronic sequelae, choroid plexa show focal calcifications, and ventricles may appear asymmetric and enlarged and be the site of fibrotic strands as linear or reticular septa, which may cause alterations in CSF flux and consequent chronic hydrocephalus. Postcontrast MRI does not show any enhancement, which is instead characteristic of the acute phase [24,32].

### 5.2. Meninges Involvement

Once the infection is in the CSF, it may reach the arachnoid and cause arachnoiditis.

**Figure 4:**
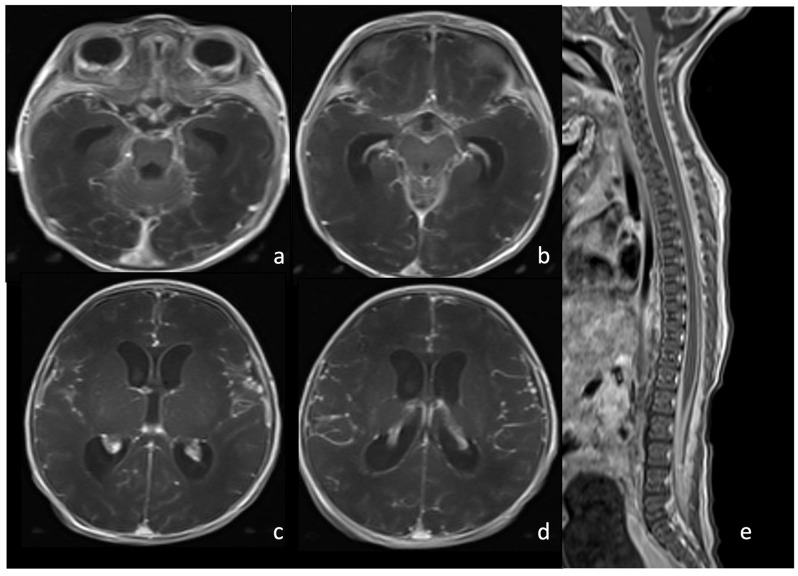
Newborn affected by *Escherichia Coli* meningitis and undergoing brain and spine MRI. Axial post-contrast T1WI of the brain (**a**–**d**) and sagittal post-contrast T1WI (**e**) of the spine show intense and diffuse pachymeningeal and leptomeningeal enhancement.

When evaluated with ultrasonographic technique, the thickness of the sulci including leptomeninges should be less than 2 mm. Among the earliest and most prevalent signs of meningitis, the increased thickness, widening, and echogenicity of the sulci indicate meningeal involvement due to the accumulation of inflammatory exudate. These findings usually fade away with meningitis treatment [24,32] (Figure 4).

MRI T2WI may show initial and subtle findings, namely an enlargement of the subdural and subarachnoid space, yet contrast-enhanced T1WI is crucial to identify a pencil-shaped leptomeningeal enhancement covering the cortical gyri and deepening in the cerebral sulci. Attention should be paid to leptomeningeal enhancement since a mild enhancement is typical of healthy meninges in asymptomatic patients and to the absence of enhancement since neonatal and early forms of meningitis do not show intense and diffuse meningeal enhancement. Dural involvement shows a thickened and enhancing dura, separated from the leptomeningeal by an enlarged subdural space, which may persist months after clinical recovery (Figure 4). FU MRI generally shows the normalization of the subarachnoid spaces and progressive enhancement decrease in the meninges during a variable time. Occasionally, dural involvement may result in persistent pachymeningeal thickening [2,8].

### 5.3. Ventriculomegaly and Hydrocephalus

Arachnoiditis leads to the formation of membranes and adhesion that can cause hydrocephalus. Hydrocephalus is characterized by an increase in CSF volume, causing ventriculomegaly due to the insufficient passage of the CSF from the production site to the liquor system. Ventriculomegaly is defined by qualitatively evaluating the ventricle dilatation and/or measuring the body of the lateral ventricle (up to 11 mm in full-term neonate) to define a mild, moderate, or severe ventriculomegaly [6,27,33].

**Figure 5:**
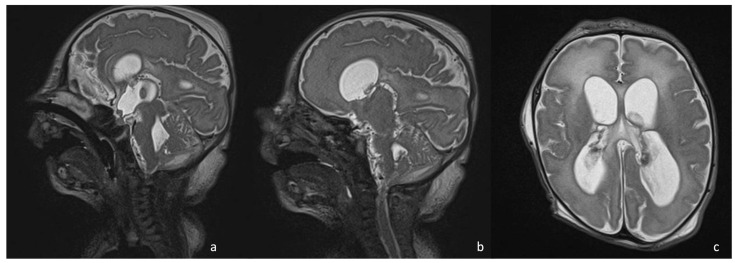
Sagittal T2 SPACE WI (**a**,**b**) showing multiple membranes and adhesions that cause hydrocephalus, visible in (**c**) (axial T2 images), in a boy with neonatal meningoencephalitis.

US, CT, and MRI may show ventriculomegaly and hydrocephalus. Particularly, MRI shows the presence of subtle membranes thanks to T2WI CISS/SPACE sequences [2,8,33,34,35] (Figure 5).

Complex hydrocephalus shows multiple encysted cavities divided by membranes or fibrotic synechiae, which may communicate with coexisting parenchymal cavitations. In fact, chronic stages are characterized by ex-vacuo hydrocephalus in patients presenting coexisting parenchymal atrophy [2,6,36].

### 5.4. Effusions and Empyema

Bacterial meningitis inflammatory exudates in the subdural space may lead to subdural effusions that may become infected and result in subdural empyemas.

Subdural effusions are fluid collections between the dura and the arachnoid, presenting the typical CSF liquor. Therefore, subdural effusions appear anechoic on US and hypodense on CT; they present a high T2WI signal and low T1WI signal, do not enhance on contrast-enhanced T1WI, and do not present a diffusion restriction on DWI/ADC.

Indirect signs of subdural effusions are brain displacement from the vault by subdural fluid causing an effacement of the subarachnoid spaces and possibly leading to a midline shift, compression of the ipsilateral ventricle, and dilatation of the contralateral ventricle [6]. Commonly, treating meningitis leads to the progressive resorption of subdural effusions [3,33,37,38,39].

Subdural empyemas are suppurative collections in the subdural space between the dura mater and the arachnoid, which may be the result of a subdural effusion infection. Most of the subdural empyemas are located in the convexity (50%) and falx (20%), with a typical crescentic shape [37,40].

At ultrasound examination, subdural empyemas appear as inhomogeneous hypoechoic collections or anechoic collections with internal echoes characterized by a crescentic shape. Rarely, they may show hyperechoic fibrous strands in case of internal septations [6].

CT shows an extra-axial iso- to hyperdense collection with a falciform shape and a marked meningeal enhancement.

MRI optimally depicts the features of subdural empyemas, seen as crescent-shaped extra-axial collections, which are limited by dural reflections and surrounded by enhancing meninges. Since subdural empyemas are collections of infected material, they appear inhomogeneously iso- or hyperintense to CSF on T2WI; hyperintense to CSF on T1WI with no water suppression on FLAIR, which would be typical of subdural effusions; and present a diffusion restriction on DWI/ADC, which cannot be seen in subdural effusions. After contrast administration, meninges rim enhancement, caused by inflammation and hypervascularity, may coexist with internal enhancing septations. Subdural empyemas favor venous thrombosis, which may require a CE-MRV for a proper evaluation [3,33,37,38,39].

**Figure 6:**
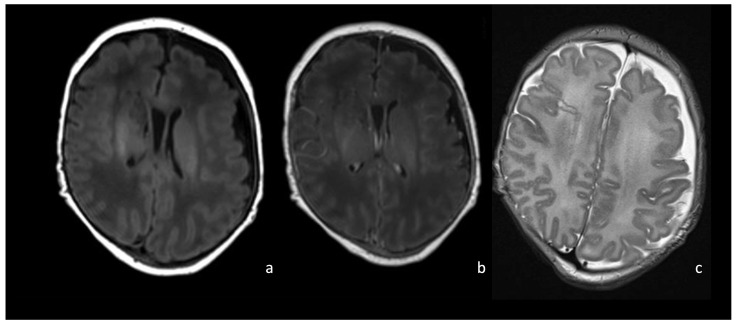
MRI of a newborn affected by *Group B Streptococcus* meningoencephalitis and presenting with left frontoparietal subdural effusion appearing hypointense on T1WI (**a**) and hyperintense on T2WI (**c**) with no appreciable contrast enhancement on post-contrast T1WI (**b**).

**Figure 7:**
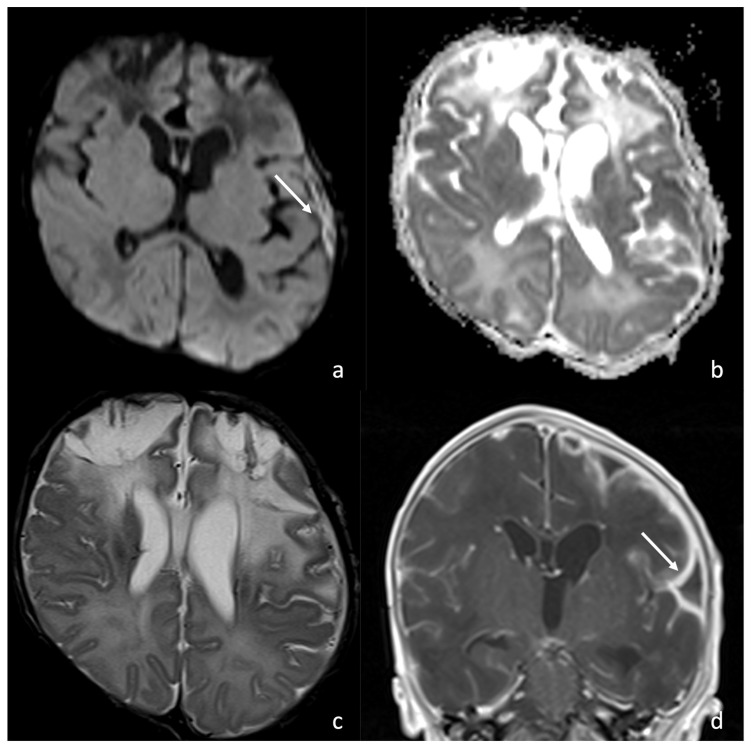
MRI of a newborn affected by *Group B Streptococcus* meningoencephalitis and presenting with left frontotemporal subdural empyema presenting diffusion restriction on axial DWI/ADC (arrow in (**a**), (**b**)) and intense pachymeningeal and leptomeningeal enhancement on post-contrast coronal T1WI (arrow in (**d**)). T2WI (**c**) shows hyperintensity of the frontal lobes WM, the left temporo-insular WM, and the deep WM bilaterally, associated with cystic degeneration of the frontal WM, frontal cortical atrophy, and laminar necrosis.

Epidural empyemas are far rarer in meningitis compared to subdural effusions and empyema. By being between the dura mater and the vault, epidural effusions and empyemas are not limited by dural reflections but by the cranial sutures and present a peculiar shape resembling a biconvex lens. Therefore, and contrary to subdural empyema, epidural collections may cross the midline in the frontal region. The most common infection routes that may cause an epidural collection are related to the contiguous spread via infected mastoid or middle ear or paranasal sinuses, or to trauma. Moreover, congenital dermal sinuses are favoring factors for recurrent meningitis and epidural collections. Epidural empyemas appear inhomogeneously iso-or hyperintense to CSF on T2WI with evidence of inwardly displaced dura seen as a hypointense line between the collection and the brain, and slightly hyperintense to CSF on T1WI with no water suppression on FLAIR. In contrast to subdural empyemas, epidural empyemas may show variable signals on DWI and a frequently variable intensity with hyperintense components. After contrast administration, there is a strong enhancement of collection margins, yet no internal septa are usually seen in contrast to subdural empyema [3,38,41,42,43,44] (Figure 6 and Figure 7).

Epidural and subdural empyema often require surgical intervention for drainage and serial FU MRIs for the evaluation of empyema progression and resolution. The empyema resolution is characterized by the absence of the infected collection in the subdural or epidural space. Meninges may retain an increased contrast enhancement at post-contrast T1, which progressively normalizes [3,33,37,38,39].

## 6. Meningoencephalitis Stage: Neuroradiological Findings

Brain involvement is common in bacterial meningitis, with the most common presentations being parenchymal abscesses and/or infarctions. The contemporary involvement of meninges and brain parenchyma in bacterial meningitis is usually referred to as bacterial meningoencephalitis.

### 6.1. Cerebritis and Abscesses

The process of abscess formation consists of four different phases that progress into each other, starting with early cerebritis and terminating with abscess late capsule formation. Particularly, cerebritis represents the inflammation of the brain parenchyma due to bacterial infection, yet no capsule to delimitate the cerebritis from the surrounding healthy brain is present. On the contrary, brain abscess refers to the presence of an infected tissue delimited by a well-defined capsule creating a barrier between the abscess itself and the surrounding healthy brain parenchyma [2,6,39,40].

1. Early cerebritis stage (3–5 days): Bacteria infiltrate the vessels causing vessel wall inflammation and vessel necrosis, which lead to blood barrier disruption and parenchymal invasion. The resulting cerebral infection, namely the early cerebritis, is limited to a focal portion of the brain, does not present a capsule, and presents a coexisting edema. In MRI, early cerebritis is seen as an inhomogeneous and ill-defined area of hyperintensity on T2WI and hypointensity on T1WI, surrounded by edema appearing hypointense on T1WI and hyperintense on T2WI. It presents a diffusion restriction on DWI/ADC in relation to cytotoxic edema and inflammatory hypercellularity. Hemorrhagic foci present as T1WI hyperintense areas within the lesion. After contrast administration, a patchy enhancement is observed, yet no capsule may be identified. On the US, early cerebritis appears as an ill-defined area of inhomogeneous echogenicity presenting increased vascularity on Transcranial Doppler, pairing CT findings, with an ill-defined area of inhomogeneous hypodensity with inhomogeneous and patchy enhancement.
2. Late cerebritis (5–14 days): Cerebritis progressively evolves to show a necrotic core and an initial encapsulation. This stage flows into and partly overlaps with the early capsule stage since this last represents a progression with similar, yet more advanced features of the late cerebritis stage. In MRI, the late cerebritis results in a focal formation characterized by a necrotic core, appearing inhomogeneous on both T1 and T2WI, without a complete and regular contrast peripheral enhancement, yet with a defined diffusion restriction on DWI/ADC. On the US, the appearance is similar to the early cerebritis, yet the lesion appears more focal and the core starts becoming hypoechogenic, similar to CT showing a significantly hypodense core in the lesion with irregular and incomplete peripheral enhancement. Early capsule formation (14–30 days): The cerebritis is becoming an abscess since the capsule is evident, yet it is incomplete and thin and appears as a hyperintense rim on T1WI and a hypointense rim on T2WI with contrast enhancement on T1WI.
3. Early capsule formation (2 weeks to 2 months): the lesion presents diffusion restriction on DWI/ADC, mainly in relation to hypercellularity. Sonographically, the lesion presents a well-defined hypoechoic core and an incomplete hyperechoic rim. CT shows a well-defined hypodense core and an incomplete peripheral enhancement.
4. Late capsule formation (weeks to months): The parenchymal abscess presents a necrotic core, appearing hypointense on T1WI and hyperintense on T2WI with diffusion restriction on DWI/ADC. The capsule is inhomogeneously thick, appearing thicker towards the cortex and thinner towards the ventricles, appears isointense on T1WI and hypointense on T2WI, and presents an intense enhancement. On the US, the abscess presents a well-defined hypoechoic core and a complete hyperechoic rim, pairing CT that shows a well-defined hypodense core and a complete peripheral enhancement.

It is important to remember that the immune system of newborns, especially of premature newborns, is not completely mature. Therefore, abscesses tend to form in a shorter time and tend to progressively enlarge due to the absence of a complete capsule [2,8,45,46].

**Figure 8:**
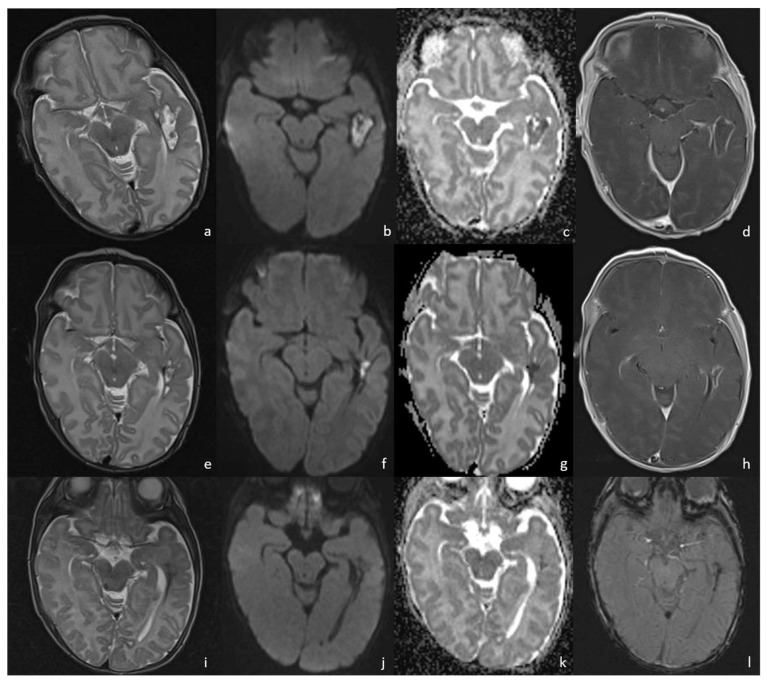
A patient affected by *Bacillus Cereus* meningoencephalitis. MRIs were acquired at 30 days, 43 days, and 63 days from birth and show the brain abscess evolution during the patient’s follow-up. At 30 days from birth (**a**–**d**), the parenchymal abscess in the left temporal lobe presents a necrotic core, appearing inhomogeneously hyperintense on T2WI (**a**) with diffusion restriction on DWI/ADC (**b**,**c**), and a thick capsule, appearing hypointense on T2WI with intense enhancement on post-contrast T1WI (**d**). At 43 days from birth, the parenchymal abscess shows a dimensional decrease and similar radiological features on T2WI (**e**), DWI (**f**), ADC (**g**), and post-contrast T1WI (**h**). At 63 days we can appreciate a hemosiderin scar appearing hypointense on T2WI (**i**) and SWI (**l**) with no diffusion restriction on DWI/ADC (**j**,**k**).

MRI is crucial to evaluate disease progression and the response to treatment (Figure 8). FU MRI shows the dimensional decrease in the lesion, which may show a liquor content appearing hyperintense on T2/FLAIR or the presence of core and parietal hemosiderin, which is better depicted as intensively hypointense on SWI. Contrast-enhanced imaging does not always help to assess treatment response since the enhancement may persist due to persistent fibrotic tissue. On the other hand, DWI/ADC optimally correlates with abscess treatment since a positive response results in the absence of a diffusion restriction and may differentiate abscess from necrotic tumors and/or cystic lesions [8,42,47,48,49].

### 6.2. Infarcts

The inflammation related to bacterial meningitis favors arterial and venous thrombosis, which may lead to cerebral infarctions. Arterial infarctions are generally related to the inflammation of the vessel wall, namely arteritis, which may complicate into arterial obstructions. In MRI, the evaluation of arteritis, which may coexist or not coexist with the vessel occlusion, may be studied with the 3D T1 TSE BB [50]. Most arterial infarctions are located along the perforator vessels of anterior and middle cerebral arteries.

**Figure 9:**
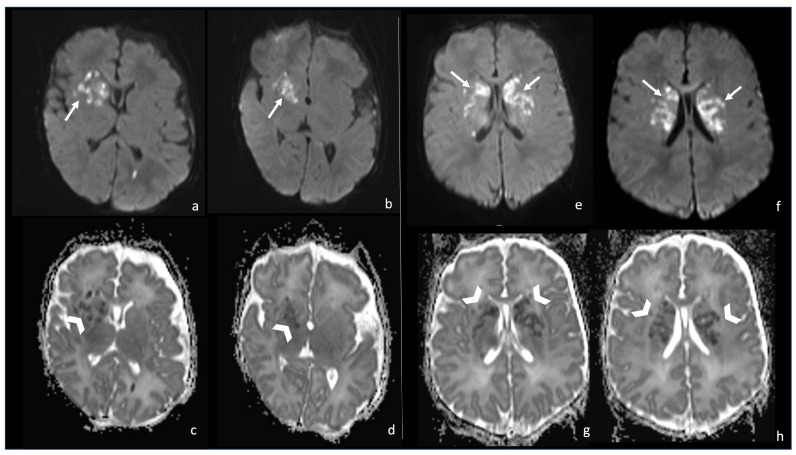
Two cases of newborns, namely a 1-month-old girl and (**e**–**h**) a 2-month-old girl, affected by *Group B Streptoccoccus* meningoencephalitis (**a**–**d**). In both cases, there are multiple punctate ischemic lesions with a massive brain involvement characterized by diffusion restriction on axial DWI sequences (arrows in (**a**,**b**,**e**,**f**)) and low ADC values (arrowheads in (**c**,**d**,**g**,**h**)).

Venous infarcts do not respect arterial vascular territories and are located in the periventricular WM in case of subependymal and periventricular veins obstruction, which is frequently associated with ventriculitis and plexitis, and/or depends on the venous sinus or vein that was obstructed. Venous clots may appear iso- or hyperintense on non-contrast T1WI depending on the age of the clot, while after contrast administration, the absence of enhancement correlates with the absence of vessel patency. Venous ischemic infarcts may not show a diffusion restriction on DWI/ADC and may easily turn into hemorrhagic infarcts, appearing hyperechoic at the US evaluation. MRI of a hemorrhagic infarct strongly depends on the timing of the infarct. The SWI sequence is very useful to depicting venous infarction. Non-contrast 2D TOF sequence or CE-MRV should be added to the protocol to evaluate the venous system in the suspicious of bacterial infection complicated with venous thrombosis [8] (Figure 9).

## 7. Peculiar MRI Patterns

In this section, we tried to highlight a few peculiar MRI findings related to some specific bacteria. However, it should be kept in mind that the following discussed findings are not pathognomonic for a specific bacterium and that all the meningoencephalitis manifestations are valid also for bacteria present in this paragraph.

### 7.1. Group B Streptococcus

*Group B Streptococcus* is one of the most common causes of neonatal meningoencephalitis, even if it is partially preventable. Maternal recto-vaginal colonization, prematurity, prolonged rupture of membranes, and maternal chorioamnionitis are known to increase the risk of neonatal infection.

At MRI examination, the pattern of multiple or diffuse infarcts is typical of *Streptococcal Meningitis* and is rarely seen in meningitis secondary to other microorganisms. Infarcts are often bilateral and basal ganglia involvement is predominant (Figure 9). In literature, authors speculate that small-vessel vasculitis or inflammatory encephalitis may be responsible for these findings [51,52,53,54,55].

### 7.2. Listeria Monocytogenes

*Listeria monocytogenes* causes purulent leptomeningitis. A typical clinical picture in early-onset neonatal Listeria infections includes maternal flu-like illness, preterm labor with intact membranes, meconium staining, perinatal asphyxia, neonatal respiratory distress, maculopapular-vesicular skin eruptions, monocytic predominance in the endotracheal aspirate, meningitis, and intraventricular hemorrhage. The involvement of the meninges and brain consists of miliary granulomas and micro-abscesses [56,57].

**Figure 10:**
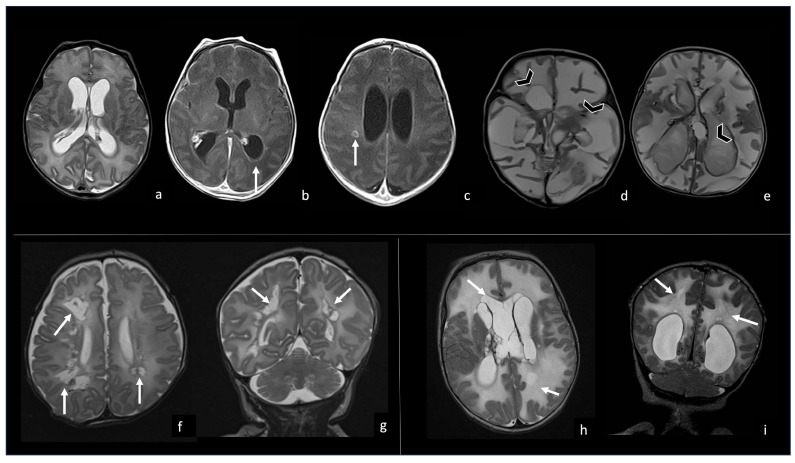
Three different cases of newborns with brain sequelae caused by *Listeria Monocytogenes* meningoencephalitis. The first case (**a**–**e**) refers to a boy of 9 days of life, who presented with meningoencephalitis characterized by ventriculitis with enlarged ventricles ((**a**), axial T2WI) with ependymal contrast enhancement (arrow in (**b**), axial post-contrast T1WI) and an encapsulated abscess in the right white matter (arrow in (**c**), axial post-contrast T1WI). At the 3-month FU, the brain MRI showed massive cystic encephalomalacia and periventricular cavitations (arrowheads in (**d**,**e**), axial T2WI). The second case (**f**,**g**) shows the 3-month FU MRI of a girl affected by listeria meningoencephalitis at 6 days of life, evolved in multiple periventricular cysts (arrows in (**f**), axial and (**g**), coronal T2WI). The third case (**h**,**i**) refers to a boy who presented with *Listeria* meningoencephalitis at 5 days of life, whose 2-month FU MRI showed encephalomalacia with multiple cysts (arrow in (**h**), axial and (**i**), coronal T2WI).

A peculiar aspect of *Listeria Monocytogenes* meningoencephalitis is the chronic evolution in cystic encephalomalacia and periventricular cavitations that are more often present in the FU MRI of meningitis caused by this bacterium than others [56] (Figure 10).

### 7.3. Gram-Negative Bacteria and Abscesses

**Figure 11:**
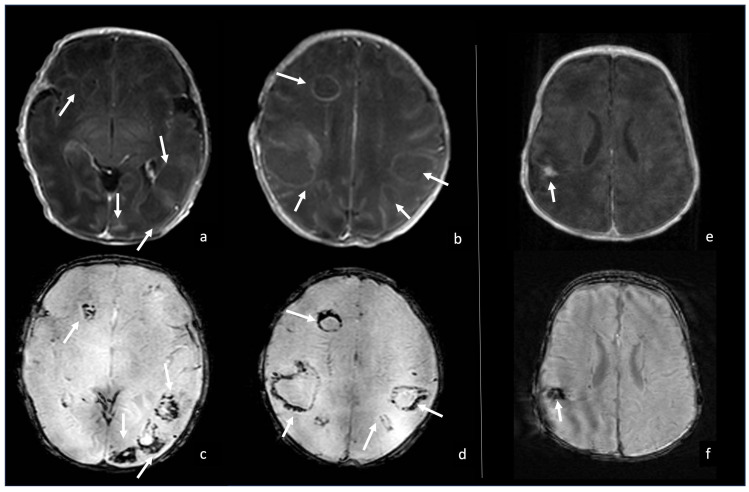
Two cases of neonatal *Klebsiella* meningoencephalitis abscess characterized by hypointense capsule on SWI due to macrophages and free radicals deposits. Case one (**a**–**d**) refers to a 5-day-old boy with multiple abscesses in bilateral white matter, whose thin capsule appears slightly visible on post-contrast axial T1WI (arrows in (**a**,**b**)) and optimally visible as a thick hypointense capsule on axial SWI (arrows in (**c**,**d**)). Case two (**e**,**f**) refers to a 1-month-old girl with a parietal abscess characterized by peripheral contrast enhancement (arrow in (**e**), axial post-contrast T1WI) and a thick hypointense capsule on SWI (arrow in (**f**), axial SWI).

In newborns, gram-negative bacteria (*Citrobacter, Proteus, Pseudomonas, Serratia, Klebsiella*) are often responsible for multiple abscesses as they have the propensity to invade nervous tissue and cause necrotizing vasculitis. Characteristically, the abscess capsule is hypointense on SWI images. This appearance should not be misdiagnosed as a hemorrhage or hemosiderin since it represents the presence of paramagnetic free radicals released by macrophages arriving at the site of infection [58,59] (Figure 11).

### 7.4. Gas-Producing Bacteria and Pneumocephalus

**Figure 12:**
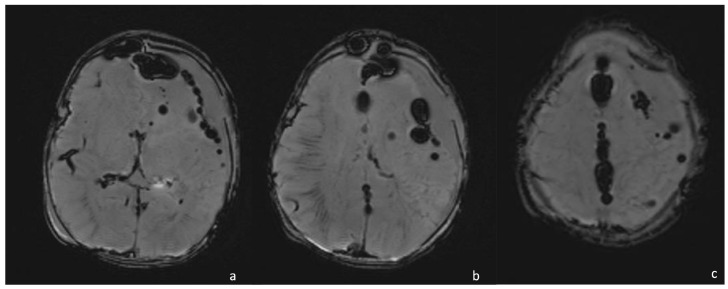
A 15-day-old boy presenting with pneumocephalus caused by *Proteus* meningoencephalitis and characterized by multiple hypointense round lesions on SWI ( (**a**–**c**), axial SWI) representing air in the intra- and extra-axial spaces.

Pneumocephalus is a rare complication of bacterial meningitis, usually occurring in the case of sepsis, caused by gas-producing bacteria such as *Clostridium Perfringens, Bacteroides Fragilis, Escherichia Coli, Klebsiella Aerogenes, Enterobacter Cloacae, Citrobacter, and Proteus*. Pneumocephalus refers to the presence of gas in the intracranial compartment. On MRI, identifying the presence of air is challenging. Gas appears markedly hypointense in all sequences and should be differentiated from flow voids and blood products. Notably, CT may easily show the presence of air as focal markedly hypodense regions (nearly −1000 HU) that may be separated or confluent [42,60,61,62,63]. Rarely, it may be appreciated a tension pneumocephalus showing the so-called “Mount Fuji” sign, namely the evidence of gas causing compression and separation of the frontal lobes with the widening of the interhemispheric region mimicking the silhouette of Mount Fuji. This sign should always suggest an immediate neurosurgical evaluation to avoid dire consequences [60,64,65] (Figure 12). Neurosurgery allows for brain decompression by air drainage, which should not be appreciable in FU MRIs.

## 8. Conclusions

Bacterial meningoencephalitis in newborns is a severe pathology, possibly leading to dare sequelae. The diagnosis of neonatal meningitis and its complications is extremely difficult and requires a multidisciplinary team of pediatricians and neonatologists, laboratory physicians, and neuroradiologists. Particularly, neuroradiology has a central role in early and non-invasive meningoencephalitis diagnosis in newborns. US represents the first imaging modality thanks to the presence of open fontanels in newborns and may offer an early and quick diagnosis at the bedside without any radiation exposure. MRI is the gold standard, offers a panoramic view of the neonatal brain, and may show all the peculiar features of bacterial meningoencephalitis, being extremely useful in cases of US uncertainties. CT is mainly limited to an emergency setting due to radiation exposure, yet the acquisition is extremely rapid and may guide the neuroradiologists to the diagnosis. Knowledge of the proper imaging techniques and the information they may provide is crucial since an early diagnosis may lead to a timely and proper treatment. Future directions encompass the creation of international protocols for neuroradiological exam acquisition that may lead to increasing the quality of exams and reducing the acquisition time, and the implementation of AI in the acquisition and interpretation of the neuroradiological exams, which may lead to increasing the sensitivity and specificity of bacterial meningoencephalitis diagnosis in newborns.

## Figures and Tables

**Table 1:** The suggested MRI protocol in case of suspected neonatal meningitis and encephalitis.

MRI PROTOCOL	
**SEQUENCES**	**PARAMETERS**
**Axial TSE T2**	RT 4730 ms, ET 109 ms, ST 2.5 mm
**Coronal TSE T2**	RT 5620 ms, ET 95 ms, ST 2.5 mm
**Axial DWI**	RT 7930 ms, ET 59 ms, ST 2.5 mm
**3D T1 MPRAGE**	RT 2200 ms, ET 3.29 ms, TI 900 ms, ST 0.8 mm
**Axial SWI**	RT 27 ms, ET 20 ms, ST 1.5 mm
**ADDITIONAL SEQUENCES**	**PARAMETERS**
**Axial FLAIR**	RT 9000 ms, ET 81 ms, ST 2.5 mm
**Axial T2 SPACE**	RT 2900 ms, ET 413 ms, ST 2.5 mm

MPRAGE: Magnetization Prepared Rapid Gradient Echo Imaging; RT: repetition time; ET: echo time; ST: slice thickness; TSE: turbo spin echo; SWI: susceptibility weighted imaging; SPACE: sampling perfection with application optimized contrast using different flip angle evolution.

## Data Availability

The data are available from the corresponding author, A.G., upon reasonable request.

## Supplementary Materials

The following supporting information can be downloaded at https://www.mdpi.com/article/10.3390/biomedicines12112490/s1, Table S1: Neuroradiological Vademecum.

## Abbreviations

CSF (cerebrospinal fluid); GBS (group B streptococcus); NICU (Neonatal Intensive Care Unit); TGC (time and gain compensation); WI (weighted-imaging); FLAIR (fluid-attenuation inversion recovery); DWI (diffusion-weighted imaging); ADC (apparent diffusion coefficient); MPRAGE (Magnetization Prepared Rapid Gradient Echo Imaging); SWI (susceptibility weighted imaging); GRE (gradient-echo); MRS (MR spectroscopy); TSE (turbo spin echo); BB (black-blood); CE-MRV (contrast-enhanced MR venography); Phase-Contrast MRI (PC-MRI); RT: repetition time; TE: echo time; TI: inversion time; FA: flip angle; ST: slice thickness; TSE: turbo spin echo; SV: single voxel; TOF (time of flight); HU (Hounsfield unit); ECMO (Extracorporeal membrane oxygenation); WM (white matter), ALARA (as low as reasonably achievable).
