# Supplementary material for: Bacterial Meningoencephalitis in Newborns
Source: Biomedicines. 2024 Oct 30;12(11):2490. doi: 10.3390/biomedicines12112490 (PMC11591924; doi:10.3390/biomedicines12112490)
Supplement: Supplementary file 1 [file biomedicines-12-02490-s001.zip › biomedicines-3254363-supplementary.pdf]

Table S1 Neuroradiological Vademecum summarizes the main US, CT and MRI neuroradiological findings that characterize bacterial meningoencephalitis.

	US	MRI	CT
**Choroid Plexitis and Ventriculitis**	**Choroid plexitis:** hyperechogenic and irregular choroid plexus.<br>**Ventriculitis:** thickened and hyperechogenic ependyma with focal irregularities indicating focal ependymal loss. Endoventricular exudates show low-level internal echoes and coexist with septa causing ventricular compartmentalisation and secondary hydrocephalus. Occasionally, increased echogenicity of the subependymal tissue.	**Choroid plexitis:** choroid plexus engorgement (post-contrast T1WI).<br>**Ventriculitis:** linear enhancement of the ependyma (post-contrast T1WI).<br>Endoventricular dependent exudates are characterized by a slightly hyperintense signal of the dependent debris and purulent tissue inside the hypointense ventricles (FLAIR) showing restricted diffusion at DWI/ADC. Intraventricular septae show subtle enhancement on post-contrast T1WI.<br>Occasionally, periventricular WM may show an abnormal signal in relation to necrosis.	**Choroid plexitis:** choroid plexus engorgement.<br>**Ventriculitis:** irregularly enhancing empyema with coexistent hypodense subependymal tissue.<br>Enlargement of the ventricles, which are septated and show a sovra-liquoral content
**Meningitis**	Increased thickness (>2mm), widening and echogenicity of the sulci	Pencil-shaped leptomeningeal enhancement covering the cortical gyri and deepening in the cerebral sulci coexisting with thickened and enhancing dura (post-contrast T1WI).<br>Enlargement of the subarachnoid and subdural spaces.	Subtle meningeal involvement or normal (CECT). Dilatation of the subarachnoid and subdural spaces.
**Ventriculomegalia and Hydrocephalus**	Enlargement of the ventricular system. Complex hydrocephalus shows multiple encysted cavities divided by membranes.	Enlargement of the ventricular system. Complex hydrocephalus shows multiple encysted cavities divided by membranes, which show a subtle enhancement on post-contrast T1WI.	Enlargement of the ventricular system. Complex hydrocephalus shows multiple encysted cavities divided by membranes.
**Extracranial Effusions**	**Subdural empyema (SE):** inhomogeneous hypoechoic collections or anechoic collections with internal echoes characterized by a crescentic shape in the subdural space. Occasionally, hyperechoic fibrous strands represent internal septations.<br>**Epidural empyema (EE):** inhomogeneous hypoechoic collections or anechoic collections with internal echoes characterized by a biconvex-lens shape in the epidural space.	**Subdural empyema:** crescent-shaped extra-axial collections which are limited by dural reflections and surrounded by enhancing meninges (post-contrast T1WI). SE are iso-hyperintense to CSF on T2WI, hyperintense to CSF on T1WI with no water suppression on FLAIR, and diffusion restriction on DWI/ADC. Possible coexisting enhancing internal septa.<br>**Epidural empyema:** epidural collection with a biconvex-lens shape surrounded by enhancing margins (post-contrast T1WI). EE appears inhomogeneously iso-hyperintense to CSF with hypointense inwardly displaced dura (T2) with no water suppression on FLAIR and variable signals on DWI.	**Subdural empyema:** extra-axial iso to hyperdense collection with a falciform shape in the subdural space, surrounded by enhancing meninges. Possible coexisting internal septa.<br>**Epidural empyema:** extra-axial collection with a biconvex-lens shape in the epidural space between the dura and the calvarium.
**Cerebritis**	**Early Cerebritis:** ill-defined area of inhomogeneous echogenicity presenting increased vascularity on Color-Doppler.<br>**Late Cerebritis:** focal area characterized by a hypoechogenic core and peripheral increased vascularity on Color-Doppler.	**Early Cerebritis:** inhomogeneous and ill-defined area of hyperintensity on T2WI and hypointensity on T1WI showing patchy diffusion restriction on DWI/ADC, surrounded by oedema (hypointense on T1WI and hyperintense on T2WI) and possibly containing T1WI hyperintense hemorrhagic foci. Patchy enhancement (post-contrast T1WI).<br>**Late Cerebritis:** focal formation characterized by an inhomogeneous and hypointense necrotic core (T1 and T2WI), with irregular and	**Early Cerebritis:** of ill-defined area of inhomogeneous hypodensity with inhomogeneous and patchy enhancement.<br>**Late Cerebritis:** focal lesion characterized by a significantly hypodense core with irregular

		incomplete peripheral enhancement (post-contrast T1WI) and defined diffusion restriction on DWI/ADC.	and incomplete peripheral enhancement.
**Abscess**	**Early Capsule Formation:** the lesion presents a well-defined hypoechoic core and an incomplete hyperechoic rim with increased vascularity on Color-Doppler.<br>**Late Capsule Formation:** the lesion shows a well-defined hypoechoic core and a complete hyperechoic rim with increased vascularity on Color-Doppler.	**Early Capsule Formation:** focal formation characterized by an hypointense necrotic core (T1 and T2WI) and a thin an discontinuous peripheral capsule appearing hyperintense on T1WI and hypointense on T2WI with defined and incomplete peripheral enhancement (post-contrast T1WI). Diffusion restriction on DWI/ADC.<br>**Late Capsule Formation:** focal lesion with a necrotic core (hypointense on T1 and hyperintense on T2) with diffusion restriction on DWI/ADC, and an inhomogeneously thick capsule (isointense on T1WI and hypointense on T2WI) with intense enhancement (post-contrast T1WI).	**Early Capsule Formation:** focal lesion characterized by A well-defined hypodense core and an incomplete peripheral enhancement.<br>**Late Capsule Formation:** lesion characterized by A well-defined hypodense core and a complete peripheral enhancement
**Infarcts**	Mostly **arterial infarcts** as hypoechoic lesions without Color-Doppler signal coexisting with loss of gyral morphology. Hemorrhagic transformation, common in **venous infarcts**, appears as hyperechoic.	**Arterial infarct:** focal area of diffusion restriction on DWI/ADC, hyperintensity on FLAIR/T2WI and hypointensity on T1WI in arterial territory, coexisting with non-patent vessel on post-contrast T1WI.<br>**Venous infarct:** focal area of hyperintensity on FLAIR/T2WI and hypointensity on T1WI in a non-arterial territory, coexisting with non-patent vessel/sinus on post-contrast T1WI. Variable diffusion restriction on DWI/ADC, frequent hemorrhagic transformation on SWI.	**Arterial infarct:** loss of gray and white matter differentiation and sulcal effacement evolving to a focal and defined area of hypodensity in an arterial territory.<br>**Venous infarct:** focal and defined area of hypodensity in a non-arterial territory, frequently hyperdense in case of hemorrhagic transformation. CECT shows a non-patent vessel/sinus.
**Pneumocephalus**	Challenging to identify.	Challenging to identify, air may appear as markedly hypointense in all sequences.	Air appears as separate or confluent areas of markedly hypodensity (nearly -1000HU).

WI: weighted-image; WM: white matter; CECT: contrast-enhanced CT; SE: Subdural empyema; EE: Epidural empyema; HU: Hounsfield Unit
